# Application of Gradient-Dependent Optimal Interpolation in Fishery Analysis of Neon Flying Squid (*Ommastrephes bartramii*) in the Kuroshio–Oyashio Confluence Region

**DOI:** 10.3390/ani13213425

**Published:** 2023-11-05

**Authors:** Chunling Zhang, Manman Cui, Wei Yu, Bilin Liu

**Affiliations:** 1 College of Marine Science, Shanghai Ocean University, Shanghai 201306, China; clzhang@shou.edu.cn (C.Z.); manmcui@163.com (M.C.); 2 Key Laboratory of Marine Ecological Monitoring and Restoration Technologies, MNR, Shanghai 201306, China; 3 National Engineering Research Center for Oceanic Fisheries, Shanghai Ocean University, Shanghai 201306, China; 4 Key Laboratory of Sustainable Exploitation of Oceanic Fisheries Resources, Ministry of Education, Shanghai Ocean University, Shanghai 201306, China

**Keywords:** vertical structure, temperature and salinity, thermocline, stock assessment, fishery oceanography

## Abstract

**Simple Summary:**

This paper provides a novel approach to integrating available environmental information into fishery data using gradient-dependent optimal interpolation. Comparative verifications of observations show that the constructed subsurface temperature and salinity profiles were very consistent with the in situ observations. The data assimilation method, named gradient-dependent optimal interpolation, gave refined matching results applied to represent the relationship between squid and environmental information. Our new method represents a useful tool in the deep research of fishery oceanography.

**Abstract:**

A key issue in fishery forecasting is the collection of high-precision subsurface environmental data. A data assimilation method, named gradient-dependent optimal interpolation, was used to construct the near-real-time vertical temperature and salinity structure of a squid fishery ground based on Argo observations. The results were verified by truth-finding comparisons and applied to analyze the relationship between neon flying squid and the subsurface environment in the Kuroshio–Oyashio Confluence Region. The temperature and salinity differences between the constructed results and survey data were less than ±0.5 °C and ±0.02, respectively. Most of the relative analysis errors were less than the observational errors. Statistical analysis revealed that the most suitable temperature for squid was 18–24 °C at the near-surface (<5 m), although the squid can endure a temperature range from 11 to 12 °C at a depth of 300 m. There was an obvious thermocline in the fishery ground, with a thermocline depth of 65 m and a mean strength of approximately 0.10 °C/m. The regressive relationship between vertical temperature (thermocline parameters) and squid catch per unit effort (CPUE) followed the exponential (Gaussian) function. The most suitable salinity was 33.0–34.2 at depths shallower than 300 m.

## 1. Introduction

Compared with the surface information, many dynamic processes occurring below the surface are more important for fishery distribution. To date, there have not been many studies on the subsurface environment in fishery analysis due to the lack of real-time marine interior observations. Most existing studies rely on sporadic observations or climate data [1,2,3,4]. The Array for Real-time Geostrophic Oceanography, known as Argo [5,6], is composed of thousands of profiling floats in oceans globally. This provides tens of thousands of observation profiles per year, and Argo has become a key component of the ocean observation system. Moreover, the extension program of the Argo has deployed multiple profiling floats that sample abundant additional subsurface information [7,8,9,10]. These floats provide a huge amount of subsurface observation data for fishery research.

Neon flying squid, *Ommastrephes bartramii*, is one of the most commercially valuable cephalopod species in the global ocean today. As one group of warm-water species, they are widely distributed in subtropical and temperate waters. *O. bartramii* is highly migratory and can be found in the North Pacific Ocean, from Japan to California, and in the South Pacific Ocean, from Chile to New Zealand [11]. It is a popular food source in many countries, and its high nutritional value and delicious taste make it a highly sought-after seafood product. With its high commercial value and wide distribution, *O. bartramii* has great potential for further development and exploitation in the future [12]. The warm water Kuroshio and cold Oyashio converge between the subtropical front (STF) and the subarctic front (SAF). Eddies, jets, and filaments are frequently separated from the main streams and merge in the confluence region [13]. This Kuroshio–Oyashio confluence region (KOCR) offers important living environments for many pelagic animals, some of which are major fishery resources, including squids [14,15,16]. The production of Chinese squid fishing, at a squid-jigging fishery in the KOCR, accounts for over 80% of the total production of squid in the North Pacific. *O. bartramii* fisheries have become an important component of China’s deep-sea fisheries.

The lifetime of *O. bartramii* is short (about one year), and it has a habit of seasonal south–north migration and vertical migration day and night. Therefore, it is very sensitive to its environment [17]. The influence of the marine environment is decisive for the spatial–temporal distribution of the squid throughout their entire lifecycle. As an indicator of fishery ground, temperature is the most important factor. The sea surface temperature (SST) is usually used for signage at fishing grounds. Previous research has shown that the suitable SST for *O. bartramii* is 13–22 °C in the KOCR [18,19]. Salinity is also a basic factor of seawater that can influence fish behavior. Every kind of fish has its own salinity suffocation ranges [3]. In addition, environmental factors, such as vertical temperature structure [20], thermocline [21], and chlorophyll-a [22], play an important role in regulating the squid aggregation. The effects of environmental conditions run throughout most of their life activities due to their short life cycles.

Integrating available ocean interior observations into fishery data is a challenging but urgent task in fishery oceanography. The gradient-dependent optimal interpolation method (gradient-dependent OI) [23] can effectively extract mesoscale information from observation data. This technique has great flexibility in constructing fishery environmental data. In this study, a novel approach was applied to match Argo profile observations with fishery data and to discuss the suitable subsurface environment of the squid. The principles and formulas of gradient-dependent OI are detailed in Section 2.2; verification of the constructed results is presented in Section 3. In Section 4, the relationship between *O. bartramii* and the subsurface seawater environment in the KOCR is discussed.

## 2. Materials and Methods

### 2.1. Fishery and Environment Data

**Figure 1:**
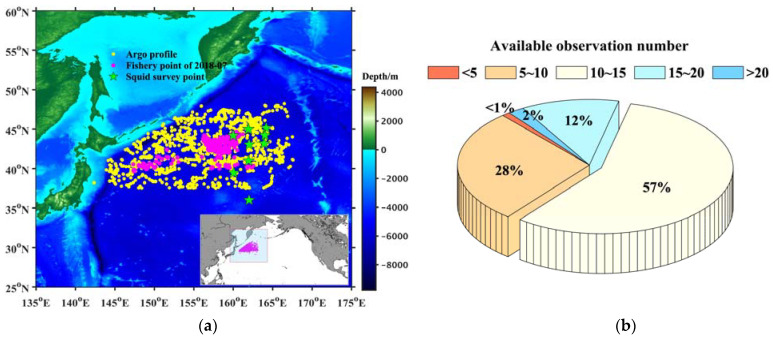
Locations of squid fishery points and the available Argo profiles during July 2018 (**a**) and the frequency statistics of available observation numbers for each fishing point (**b**).

The fishery data and commercial squid catch data were derived from the Chinese Squid-jigging Science and Technology Group of the Shanghai Ocean University. The coverage time was from 1 July 2009 to 31 November 2018. Information, such as the catch (in tons), fishing effort (the fishing time, in days), locations (longitude and latitude), and date (year and month) of the fishing operations were all included in the fishing logs. As illustrated in Figure 1a, the fishing ground of *O. bartramii* was mainly located within 140° E–170° E, 30° N–55° N in the KOCR. The fishing operations were concentrated in the months from July to November, with a total 91,843 fishery points over 10 years. The daily catch was considered as the nominal catch per unit effort (CPUE, unit: t/day) used to evaluate the abundance of the squid in this study.

The environmental survey data of fishery ground were provided by “SongHang” during a scientific investigation in the Northwest Pacific Ocean. There were nine squid fishery points (Figure 1a), with the in situ temperature and salinity profiles obtained through integrated observation by a conductivity–temperature–depth survey (SBE 911plus CTD, Sea-Bird Scientific, Washington, DC, USA) from 5 July 2022 to 22 July 2022. Their locations and dates were detailed in Table 1. These in situ observations were processed by delayed-mode quality control [24] to verify the accuracy of the constructed results.

The environmental observations adopted in this paper were Argo profiles provided by the China Argo Real-Time-Data Center (ftp://ftp.argo.org.cn/pub/ARGO/global/ accessed on 22 March 2023). Standard Argo profiles contain temperature, salinity, and pressure data, with hundreds of unequal intervals sampled at depths between 5 m and 1500 m. The profiles surrounding the squid fishery points were collected from 1 July 2009 to 31 November 2018 in the KOCR (Figure 1a). We set a valid radial of 500 km to ensure that there were more than five available Argo profiles within the radial of each fishery point. Surrounding the 91,843 fishery points, a total of 431,644 Argo profiles were used in this study to construct subsurface information of the fishery ground. All the observation profiles were processed by real-time and delayed-mode quality control [24]. The locations of fishery points of July in 2018 and their corresponding Argo profiles within the valid radius are displayed in Figure 1a. For the most part, there were at least 10 profiles within the radial distance surrounding each fishery point (Figure 1b).

### 2.2. Construction Algorithms

The construction algorithm used in this study was first developed for data assimilation [23] and applied to match the environmental observations with fishery data in this study. The method, named gradient-dependent OI, was an improved optimal interpolation (OI) based on the gradient-dependent correlation scale. The generalized analysis equation of the algorithm was as follows:

(1) $$v_{i}^{a}=v_{i}^{b}+\sum_{j}^{M}w_{ij}\delta y_{j}^{0}$$

where $v_{i}^{a}$ and $v_{i}^{b}$ are the analysis results and first guess or background value of the fishery point, with v  being the temperature or salinity. The subscript *i* denotes the number of fishery sampling points. A set of *M* observations, $y_{j}^{0}$, was available within the radial distance surrounding each fishery point, and *j* is the number of Argo profiles. $\delta y_{j}^{0}=y_{j}^{0}-H\left(v_{j}^{b}\right)$, named observational increments, were converted by the observational operator, H, from background to observations. All the available observational increments were weighted by the optimal weights, $w_{ij}$, determined by solving the following Equation (2) [25]:

(2) $$\sum_{j=1}^{M}w_{ij}\mu_{jk}+\eta_{k}w_{ik}=\mu_{ik},\;\;k=1,\;\cdots ,M$$

where η is the mean square of the relative observation errors standardized by background errors. The value of η was set to be 0.25, according to the ideal experiment from a previous study in the Pacific Ocean [23]. The subscript *j* and *k* indicate the number of available Argo profiles. The background error correlations, $\mu_{ik}$ and $\mu_{jk}$, denote the relevance of the *i*th fishery point and the *k*th observation, respectively, at two observational points, j and k. These correlations followed a Gaussian exponential function, as follows [23]:

(3) $$\mu_{ik}\;\sim \;exp\left[-\frac{{\left(x_{i}-x_{k}\right)}^{2}}{{\left(L_{\emptyset }/G_{x}\right)}^{2}}-\frac{{\left(y_{i}-y_{k}\right)}^{2}}{{\left(L_{\emptyset }/G_{y}\right)}^{2}}\right]$$

(4) $$G_{x}=1+\frac{\left|\partial v/\partial x\right|}{E\left(\left|\partial v/\partial x\right|\right)}\;,\;\;\;\;\;\;G_{y}=1+\frac{\left|\partial v/\partial y\right|}{E\left(\left|\partial v/\partial y\right|\right)}$$

where x and y represent the longitude and latitude of locations, respectively. The correlation scale, $L_{\emptyset }/G$, was determined by a Rossby radius, $L_{\emptyset }$, divided by the horizontal gradients. $L_{\emptyset }$ was typically taken as a value of 500 km multiplied by the cosine function of the latitude of the fishery point. The G parameter contained a zonal component ($G_{x}$) and a meridional component ($G_{y}$) that provided an anisotropic background error according to the horizontal gradient changes.

The real-time temperature and salinity profiles which had the same time (day) and location as the squid fishery data were constructed using the algorithm described in Equations (1)–(4). All the profiles contained 24 layers (5–1500 m) with unequal intervals and illustrated the vertical structures of the *O. bartramii* fishery ground in the KOCR.

## 3. Results

### 3.1. Verification of the Results

**Figure 2:**
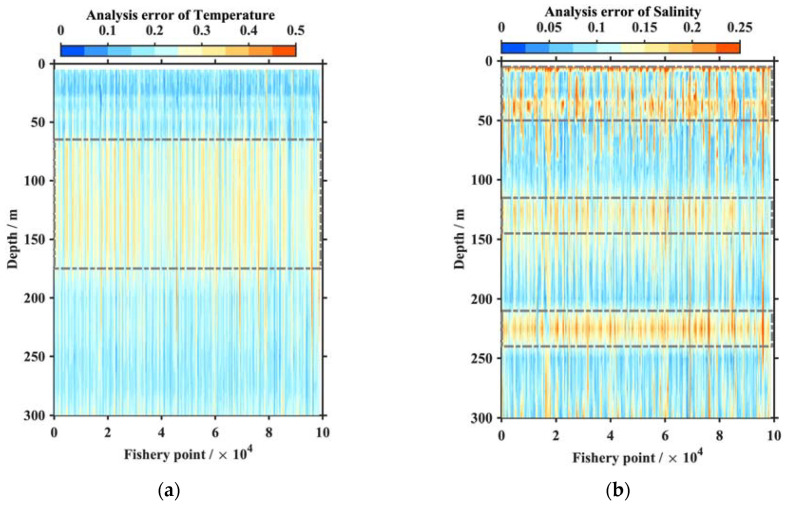
Relative analysis errors of temperature (**a**) and salinity (**b**) at each fishery point.

The optimal interpolation scheme presented in Section 2.2 provided a theoretical estimation error, with the relative analysis error given by $e_{i}=1-\sum_{j=1}^{M}w_{ij}\mu_{ij}$, standardized by the background error. The relative analysis error indicated the construction accuracy using background error as a benchmark. Figure 2 shows that the temperature analysis errors were less than half of the background error, and the salinity analysis results represented no more than 0.25 (i.e., 25% of the background error). This indicated that the temperature errors were equal to the observation errors, and salinity was closer to the true value than observation. Most of the temperature (salinity) errors were less than 0.25 (0.125) and at all the fishery points, apart from the thermocline (approximately 65–175 m for temperature) or the halocline (about 110–150 m), where vertical temperature and salinity changed rapidly. The surface- and mid-layer analysis errors of salinity exceeded 20% of background that equaled 80% of the observation errors.

**Figure 3:**
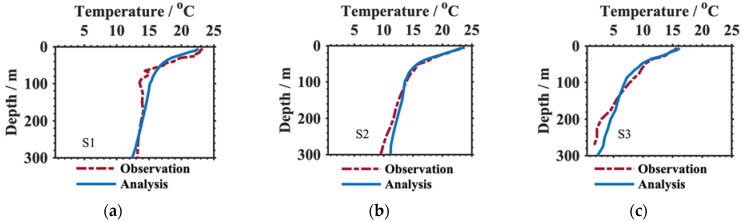

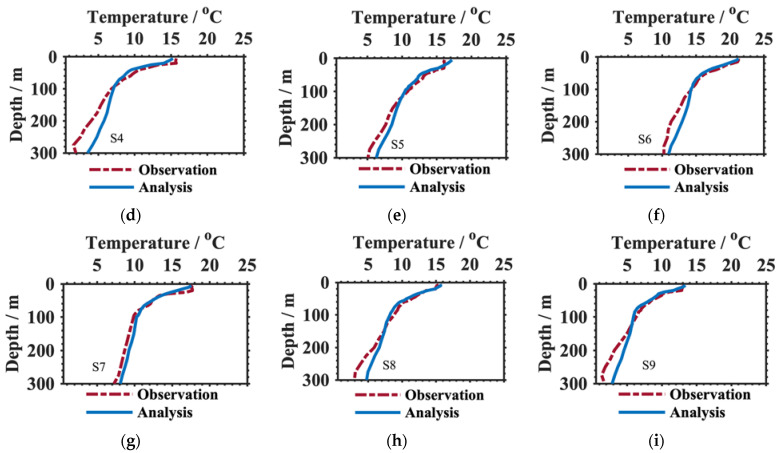
Comparison of the temperature analysis (blue solid line) and observation (red dotted line) profiles at the nine squid survey points indicated in Figure 1a at depths shallower than 300 m. (**a**–**i**) show the temperature differences at the locations S1, S2, S3, S4, S5, S6, S7, S8, S9 respectively.

**Figure 4:**
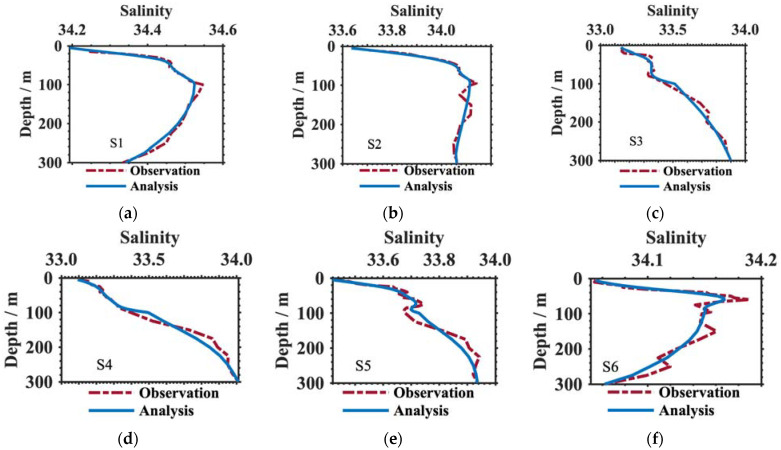

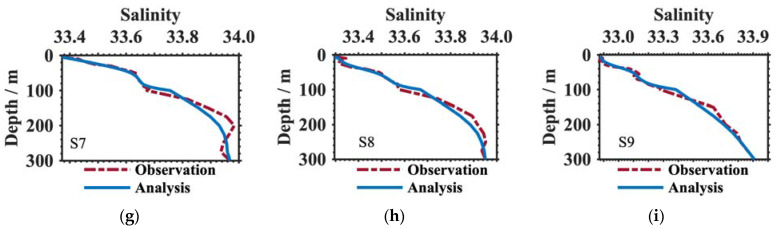
Comparison of the salinity analysis (blue solid line) and observation (red dotted line) profiles at the nine squid survey points indicated in Figure 1a at depths shallower than 300 m. (**a**–**i**) show the salinity differences at the locations S1, S2, S3, S4, S5, S6, S7, S8, S9 respectively.

The nine squid survey profiles of temperature and salinity presented in Figure 1a and Table 1 (S1–S9) were taken as the true values at the fishery points; we constructed their analysis profiles using Equations (1)–(4). Then, the analysis results were verified by comparing their observed profiles. Notably, the observed profiles were filled, and the deficiencies were corrected by data quality control [24]. Figure 3 and Figure 4 clearly display the temperature and salinity comparisons at depths of 5–300 m, respectively. Demonstrably, the constructed results (blue lines) had good consistency with the true values (denoted by the red lines), especially at the near-surface (5–100 m), which was almost coincident with the observation profiles at each point. As for the temperature (Figure 3), there was an obvious thermocline at the constructed results between tens of meters and two hundred meters. The thermocline gradients at the observation profiles were weak. Additionally, there was a slight difference between the temperature results and the true values at depths of 200 to 300 m, although these differences were no more than 0.5 °C. This might be related to the stability of the Argo temperature sensor, which is better than that of portable conductivity, temperature, and depth (CTD) profilers. The same features were also represented in the salinity profiles. The vertical changes in both kinds of salinity profiles were similar to each other, but the analysis results were smoother and more stable than the observations. The salinity differences between analysis results and observations were approximately 0–0.02.

### 3.2. Application and Analysis

**Figure 5:**
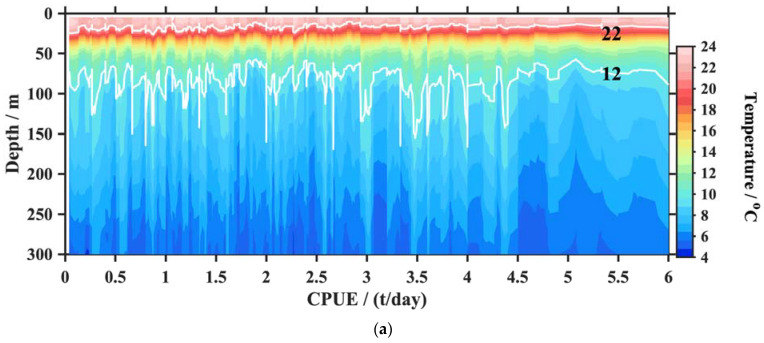

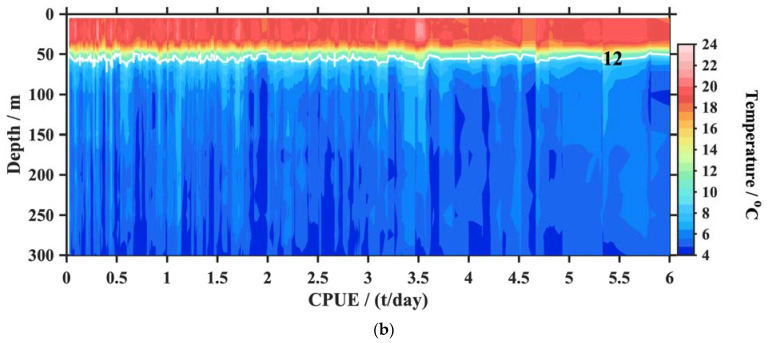
Temperature sections corresponding to different squid CPUE in August (**a**) and October (**b**). The white lines indicate the temperature contour with a value of 8 and 16 °C.

**Figure 6:**
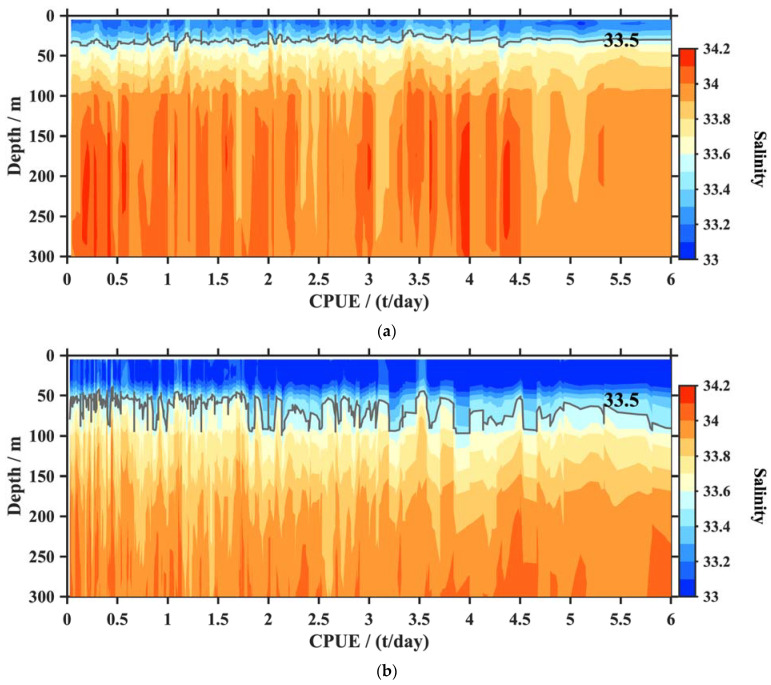
Salinity sections corresponding to different squid CPUE values in August (**a**) and October (**b**). The white lines indicate the salinity contour with a value of 33.5.

**Figure 7:**
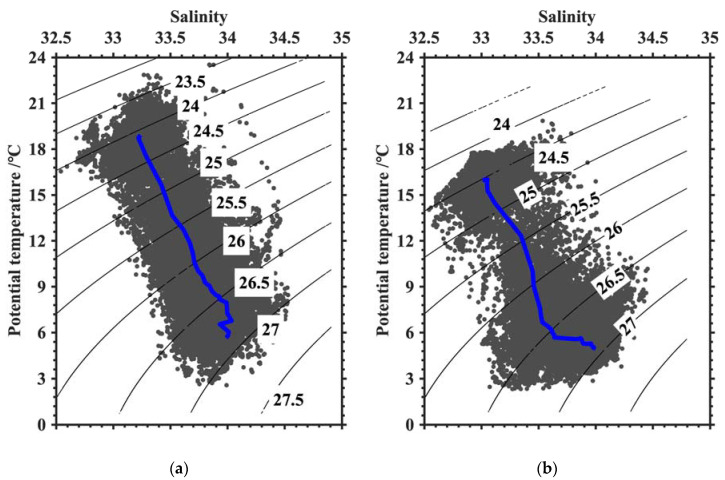
Water mass properties of the squid fishery ground in August (**a**) and October (**b**). The black lines indicate the potential density (unit: kg/m^3^). The blue lines are the mean T–S plots.

The constructed temperature and salinity profiles corresponding to 91,843 *O. bartramii* fishery points were applied to analyze the relationship between the squid CPUE and the oceanic environmental factors. Most of the squid catch values corresponding to these profiles were concentrated in 1–6 t: <2 t (21%), 2–4 t (47%), 4–6 t (28%), and >6 t (4%). To illustrate the vertical characteristics in different seasons, we selected two months with more fishery points, namely August and October, to sample profiles and interpolate the temperature and salinity of each water layer to a CPUE of 1–6 t. Their sample sizes were 19,856 and 17,119, respectively. The temperature and salinity sections for the squid CPUE changes are displayed in Figure 5 and Figure 6. The suitable temperature and salinity property are statistically represented by the water mass, as shown in Figure 7.

Figure 5 displays the general characteristics of the temperature distribution corresponding to different CPUEs. At the squid fishery ground, the near-surface temperatures were approximately 24 °C in August and 20 °C in October. The temperature remained warm (>12 °C) up to a depth of 100 m in August and 50 m in October. At a depth of 150 m, the temperature decreased significantly with a value of lower than 8 °C. There was an evident thermocline corresponding to each CPUE value. The thermocline depth in August (−80 m) was deeper than that of October (−50 m) due to the mixed strengthening effect in the autumn–winter seasons. When the CPUEs were larger than 4.5 t, the temperature isopleths tended to be smooth. In contrast, the temperature fluctuated greatly with the CPUE change when the values were small. The vertical temperature characteristics at the upper mixed layer (<30 m in Figure 5a and <50 m in Figure 5b) are clearly displayed. Many fishery points with higher CPUEs exhibited more stable temperature curves. Otherwise, the temperatures had large fluctuations at the squid fishery points with lower CPUEs. At all fishery points, the vertical temperature change showed a value of 4–24 °C at a depth of 5–300 m in August and 4–20 °C in October.

Salinity was also an important environmental factor. The superposition analysis suggested that the relationships between the squid CPUE and seawater salinity was not stationary (see Figure 6). Overall, the salinities derived in August were higher than those found in October. The salinities increased further downward. The near-surface salinity was less than 33.5 at depths lower than 50 m. The salinity was concentrated at 33.5–33.9 for August and 33.3–33.8 for October between 50 and 100 m. Then, the salinities increased to 34.0 below 100 m in August and remained at a lower value of about 33.8 in October. Most fishery points had mean salinity values of 33.0–34.2 at depths of 5–300 m. The fishery points with higher CPUEs (>4.5 t) exhibited vertical minor salinity changes. In contrast, the salinity profiles showed an obvious vertical difference when the CPUEs were less than 4.5 t.

As for the water mass property indicated in Figure 7, the T–S (temperature–salinity) scatter relationships were relatively discrete in the squid fishery ground. The water mass had certain fluctuations in properties especially in October. However, Figure 7 displays the clear temperature and salinity characteristics of the fishery points. At near-surface layers, high temperature (>18 °C) and low salinity (<33.5) features corresponded to the isopycnal with a value of 24.5 kg/m^3^. In the deep water, the density increased gradually due to temperature decreasing and salinity increasing. Along the isopycnal with value of 26.5 kg/m^3^, the temperature of fishery points reduced to 6 °C with a salinity of about 33.8. The water mass property of the fishery ground showed the seasonal differences in deep water (density was larger than 26.5 kg/m^3^).

## 4. Discussion

### 4.1. The CPUE and Vertical T/S

Similar to most animals swimming at upper layers, *O. bartramii* moves vertically day and night. They generally inhabit depths of 200–300 m in the day and swim to shallower water (<50 m) at night [26]. Their habitat depths depend on the vertical temperature and salinity structures. Most CPUEs in the fishery data were approximately 2–6 t in this study. Only 5% of squid fishery points had a CPUE that exceeded 6 t. According to their weights, the squid CPUEs were divided into four groups: <2 t, 2–4 t, 4–6 t, and >6 t. Table 2 and Table 3 detail the four CPUE clusters and their suitable temperature or salinity ranges, respectively.

At the near-surface (<5 m), the suitable temperatures were 20.6–23.8 °C in August and 18.9–21.7 °C in October, respectively. Compared with the October dataset, the temperatures from August were slightly warmer, especially at depths of 5–150 m, as evident in Table 2. At this depth, the temperatures dropped by more than 10 °C. At a depth of 300 m, the most suitable temperatures were 4–8 °C. In both seasons, the fishery points with larger CPUEs (4–6 t) exhibited a small temperature change at the same depth. When the CPUEs exceeded 6 t, the suitable temperatures were concentrated on a very small range. In contrast, the lower CPUE clusters (0–4 t) demonstrated a considerable temperature change at every depth. Regarding the vertical temperature changes, similar characteristics were represented in different CPUE clusters. The temperature differences between the surface and 300 m were more than 15 °C for each CPUE cluster. These were consistent with previous research results [21].

The squid migrate to the upper ocean, where salinity is a key environmental factor [27]. The salinities derived in August were higher than those found in October, as presented in Table 3. At the near-surface, most of the fishery points had salinity values of 33.0–33.7. That was lower due to the mixing of the water. Table 3 shows that the suitable salinities were at higher values, exceeding 33.5 at a depth of 150 m. At a depth of 300 m, the most suitable salinities were 33.9–34.2 in August and 33.7–34.1 in October. The salinities in August were higher than those in October corresponding to the same depth. Additionally, the vertical and horizontal salinity changes were slow when the CPUEs exceeded 4 t. When the salinities were in the range of 33.1–34.0, the CPUEs increased. However, the CPUEs were lower with a salinity larger than 33.0 or smaller than 34.2.

**Figure 8:**
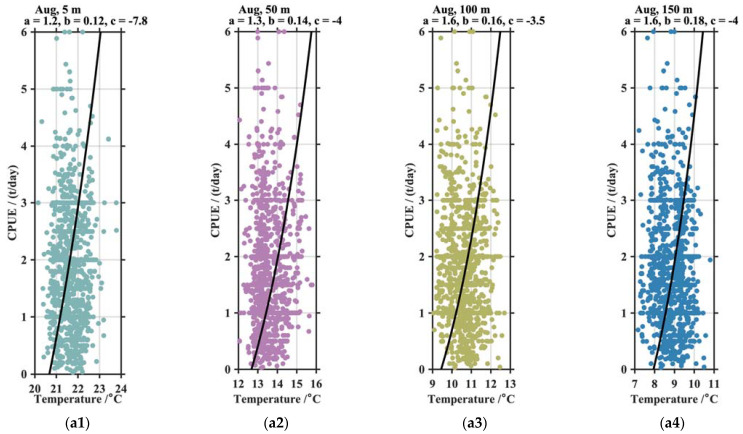

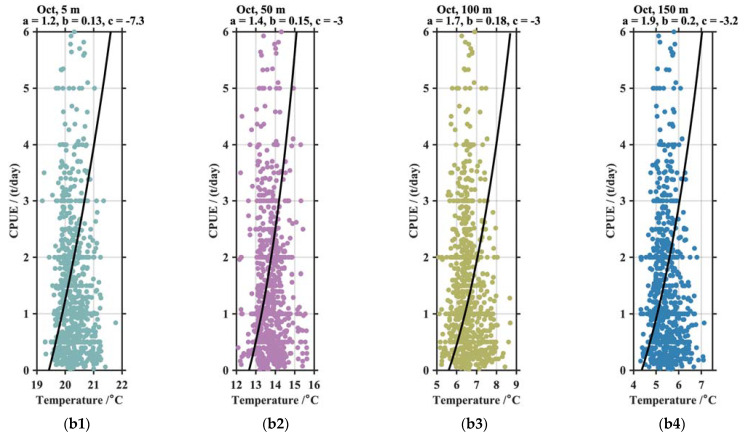
The scatter and regressive analysis of the squid CPUE and temperature in August (**a1**–**a4**) and October (**b1**–**b4**) at different depths. The green, purple, yellow, and blue dots represent statistical results of 5 m, 50 m, 100 m, and 150 m respectively. The black lines indicate the curve fitting of function $C=a\cdot e^{-bT}+c$

The distribution of fishery grounds was closely related to the vertical temperature structures. The suitable temperatures were significantly different at various depths. The scatter distribution indicated that the regressive analysis of the vertical temperatures (denoted by T in Figure 8) and CPUEs (denoted by C in Figure 8) fitted an exponential function of $C=a\cdot e^{bT}+c$ at each water layer. Both the correlation coefficients, namely a and b, were positive values at any depth. The CPUEs increased following an exponential function when the vertical temperature increased within their suitable temperature ranges. Compared with August, the scatters in October were more concentrated, although the suitable temperatures were demonstrably lower.

### 4.2. The CPUE and the Thermocline

**Figure 9:**
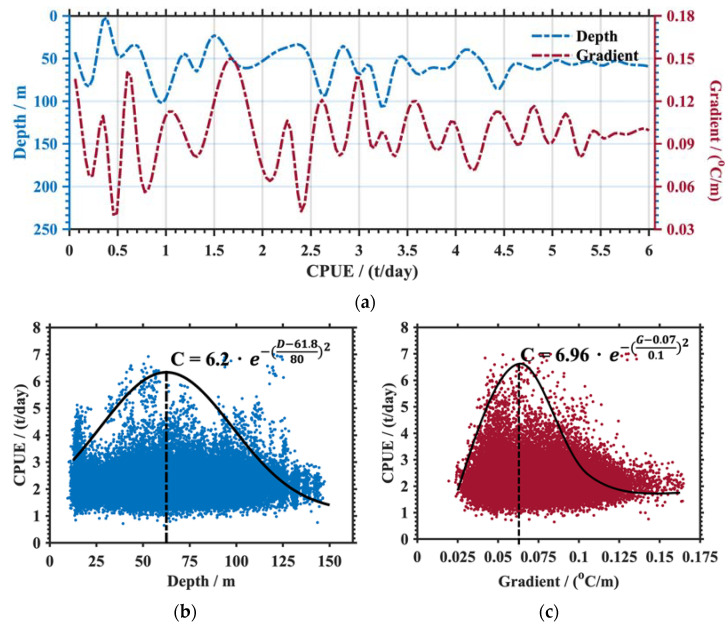
Statistics of the thermocline parameters and CPUE. (**a**) The curves of thermocline depth (blue line) and gradient (red line) corresponding to different CPUEs. (**b**,**c**) The scatters of thermocline depth (blue dots) and gradient (red dots) for CPUE. The black solid lines indicate Gaussian curve fitting; black dotted lines are the locations of the symmetry center.

As a natural barrier, the thermocline influenced the migration of the squid up and down [21]. These parameters, namely the thermocline depth (abbreviated as Depth or D in Figure 9) and temperature gradient that indicated the thermocline strength (abbreviated as Gradient or G in Figure 9), were calculated using the maximum angle method in this study [28]. Firstly, the thermocline parameters were derived from each Argo temperature profile surrounding the fishery point. They were then merged with the fishery points by the gradient-dependent OI detailed in Section 2.2. Here, only the seasonal thermocline located at the upper depths was considered.

Combining Figure 1a and Figure 5, the fishery points were mainly located in the KOCR where the thermocline was clear in summer and autumn. Figure 9a shows that the mean thermocline depth (upper boundary) was about 65 m. At points with lower CPUEs, the thermocline depths exhibited significant fluctuation. When the CPUE exceeded 4 t, the thermocline depth curve was smooth. At all of the fishery points, their corresponding thermocline temperature gradients were larger than 0.05 °C/m. Additionally, the maximum thermocline gradient was approximately 0.15 °C/m, with a mean value of 0.10 °C/m. Similar to the thermocline depth, the gradient curve was more stable when the CPUE increased. However, the strength of the thermocline was not simply proportional to the CPUE. As indicated in Figure 9b,c, scatters of CPUE and thermocline parameters fitted a certain Gaussian function. The CPUE first increased and then reduced with the thermocline parameters intensifying. When the thermocline depth was 61.8 m, the CPUE reached a peak of about 6.2 t with a variance value of 8 m. The CPUE had a statistical maximum value of 6.9 t corresponding to a thermocline gradient of 0.07 °C/m, whose variance was 0.1 °C/m. In other words, the extremely strong thermocline (a drastic change in temperature) would restrict the vertical movement of *O. bartramii*.

## 5. Conclusions

A novel application of the data assimilation method was proposed to integrate Argo observations into fishery points in this study. The effectiveness of gradient-dependent OI in constructing a real-time interior environment for *O. bartramii* fishery points was verified with more than 90,000 locations spanning 10 years in the KOCR. Most of the constructed temperature and salinity profiles exhibited analysis error less than the observational errors. The analysis results represented differences of less than ±0.5 °C for temperature and 0.02 for salinity by truth-finding comparisons. The number of available observations and their distance from the fishery point were important for the construction accuracy. The gradient-dependent OI will become increasingly suitable for widespread usage because of the rapid development of Argo profiles.

During 2009–2018, the most suitable temperatures for the squid were 20.6–23.8 °C in August and 18.9–21.7 °C in October. The temperatures reduced as the depth increased. There are seasonal differences in suitable temperatures. Change in the suitable temperatures may be related to the squid life stage. The suitable salinities changed to 33.2–34.6 within 300 m. The thermocline showed strong correlation with the CPUEs. The CPUEs of *O. bartramii* increased following an exponential function of vertical temperatures, and they reached peak values with a thermocline of 61.8 m and a thermocline gradient of 0.07 °C/m in the curve fitting of Gaussian functions.

The application carried out in this paper indicated that the gradient-dependent OI is effective in fishery oceanography research. It is common knowledge that fishery activities depend on complex environmental effects. Thereby, many more types of near-real-time environmental information could be constructed by gradient-dependent OI in future with the growth of Argo observation profiles containing further factors, including dissolved oxygen and chlorophyll.

## Figures and Tables

**Table 1:** The information of nine squid fishery points.

	S1	S2	S3	S4	S5	S6	S7	S8	S9
Longitude	162.0° E	160.0° E	160.0° E	161.9° E	162.0° E	161.9° E	163.9° E	164.0° E	164.2° E
Latitude	36.0° N	39.5° N	44.2° N	44.9° N	43.0° N	41.0° N	43.2° N	44.2° N	45.1° N
date	5 July 2022	9 July 2022	13 July 2022	14 July 2022	15 July 2022	16 July 2022	20 July 2022	21 July 2022	22 July 2022

**Table 2:** The suitable temperatures (°C) for the squid at different depths for each CPUE cluster.

	August				October			
	5 m	50 m	150 m	300 m	5 m	50 m	150 m	300 m
<2 t	20.6–23.8	12.1–15.7	7.6–10.8	4.5–7.5	19.8–21.7	11.4–17.9	4.0–6.8	4.0–5.7
2–4 t	21.5–23.5	12.2–15.3	7.7–10.3	4.2–8.1	19.0–21.3	11.6–17.6	4.0–7.2	4.0–6.3
4–6 t	22.2–23.4	12.4–14.6	7.5–9.2	4.9–7.3	18.9–20.9	12.1–17.0	4.0–5.9	4.0–6.1
>6 t	23.4–23.6	14.1–14.7	7.7–8.4	5.1–6.0	19.3–19.9	12.5–15.8	5.3–6.5	5.1–5.7

**Table 3:** The suitable salinities for the squid at different depths for each CPUE cluster.

	August				October			
	5 m	50 m	150 m	300 m	5 m	50 m	150 m	300 m
<2 t	33.1–33.6	33.4–33.7	33.8–34.2	34.0–34.2	33.0–33.5	33.2–33.6	33.7–33.9	33.7–33.9
2–4 t	33.1–33.4	33.6–34.0	33.8–34.3	33.2–34.2	33.0–33.7	33.4–33.8	33.7–33.9	33.7–33.9
4–6 t	33.1–33.2	33.6–33.7	33.8–34.2	34.0–34. 2	33.1–33.2	33.1–33.6	33.6–33.7	34.0–34.1
>6 t	33.1–33.2	33.5–33.6	33.8–33.9	34.0–34.1	33.0–33.1	33.3–33.4	33.7–33.8	33.9–34.0

## Data Availability

Three categories of data used to support the results of this study are included in the article. *O. bartramii* fishery data were provided by the Chinese Squid-jigging Science and Technology Group. The fishery data are available from the National Data Centre for Distant-water Fisheries of China (DCFC) (https://hyxy.shou.edu.cn/sddwf/12862/list.psp accessed on 15 November 2022). Argo float data were collected and made freely available by the China Argo Real-time Data Center (ftp://ftp.argo.org.cn/pub/ARGO/global/ accessed on 22 March 2023). The climatic environmental data, World Ocean Atlas 2018 (WOA18) gridded data are available from the Ocean Climate Laboratory of NODC (https://www.nodc.noaa.gov/OC5/woa18/ accessed on 18 August 2023).
